# Professional Responsibility: Conceptual Rescue and Plea for Reform

**DOI:** 10.1093/ojls/gqab010

**Published:** 2021-06-18

**Authors:** Sylvie Delacroix

**Keywords:** professional responsibility, vulnerability, duty to consult, Montgomery, risk disclosure, sense of self

## Abstract

For as long as knowledge asymmetry continues to be deemed the defining characteristic of the lay-professional relationship, the courts’ delineation of obligations meant to address lay vulnerability will too frequently end up compounding the layperson’s non-epistemic, ‘sense of self’ vulnerability. The proposed re-conceptualisation of professional responsibility calls for reform on several fronts: among these, an expanded ‘duty to consult’ (beyond do-not-resuscitate-orders) is uniquely placed as a justiciable criterion capable of addressing such a situational, ‘sense of self’ vulnerability.

## 1. Introduction

As a sociological concept, the professions’ remit is necessarily uncertain: it is constantly renegotiated as more and more occupations strive to be recognised as a ‘profession’. As a legal and ethical concept, ‘professional responsibility’ is often understood in a similarly loose fashion. Most are content to associate professional responsibility with some rather vague ‘public service’ dimension that could possibly warrant extra duties. The cost of this conceptually haphazard delineation of professional responsibility is insidious. While some domains—such as education—are stuck with inadequate responsibility practices, novel occupations that ought to be held to different responsibility standards are treated in the same way as generic service providers.

The roots of this haphazard delineation can be traced to several factors: historically, the concept of professional responsibility has never really recovered from its entanglement with some ill-advised ‘professionalism’ rhetoric. According to the latter, the professions’ special status (both in terms of responsibility and privileges) would take its roots in the presumed epistemic and character superiority of its members. It is the combination of this rhetoric with the frequent conceptualisation of responsibility by sole reference to the responsible agent’s capacities that has eroded the credibility of professional responsibility as a concept worthy of rigorous scholarly attention.

This article proceeds from a ‘conceptual rescue’ of professional responsibility. Like many rescue missions, it entails sacrifices. The scope of professional responsibility as it emerges from this article is notably different from the ever-changing, sociologically delineated scope of those occupations deemed ‘professional’. Why? Because this article ties the rationale for professional responsibility to the situational, ‘sense of self’ vulnerability that is concomitant with many—but not all—of the circumstances that prompt recourse to services deemed ‘professional’. The struggle to preserve one’s health or one’s social standing and recognition (which sudden poverty, job dismissal, prosecution or illness can all endanger) is likely to jeopardise a person’s ability to minimally ‘own’ the way she projects herself, both socially and through her body. This article highlights the way in which the specificity of professional responsibility only makes sense if it is deemed to find its roots in the extent to which, in such circumstances, a professional may either alleviate or contribute to this ‘sense of self’ vulnerability.^1^

Now you might ask: what about all the rather mundane circumstances that can prompt recourse to services also deemed ‘professional’? My consulting a lawyer in a bid to acquire a company is unlikely to ever imperil the continued construction of my ‘sense of self’. In such cases, the responsibility at stake is no different from that of all expert service providers. The only thing a commercial lawyer’s responsibility has in common with that of an oncologist or criminal lawyer is that their presumed epistemic superiority (just like a scuba diver instructor’s) warrants special disclosure obligations. The similarity ends there. The problem is that the current, lax understanding of professional responsibility encourages a lowest common denominator approach. Not only does this approach provide no rationale for distinguishing between the responsibility that arises from a commercial lawyer advising me on a company acquisition and that of a GP helping me deal with a chronic illness. This lax understanding is also at the root of the fact that professional obligations designed to address lay vulnerability (such as disclosure obligations) frequently end up compounding the layperson’s non-epistemic, ‘sense of self’ vulnerability described above.

To remedy the above situation, this article proceeds in four steps. Section 2 starts by articulating the difference between kinds of vulnerabilities. The delineation of the obligations that stem from professional responsibility typically only take into account ‘inherent’^2^ forms of vulnerabilities: it is because we are fragile, enmattered^3^ beings who need to rely on others’ expertise to navigate today’s world that we resort to all sorts of service providers. These corporeal and epistemic forms of vulnerability are inherent in that they cannot be helped, and are not context-dependent. In contrast, the ‘sense of self’ vulnerability concomitant with many of the circumstances prompting recourse to a professional stems from the extent to which my social identity is entangled with my professional, familial, legal and health status. Section 2B develops an account of professional responsibility that is grounded in the situational, sense of self vulnerability described above and highlights the way in which it differs from the current piecemeal approach to professional responsibility.

Section 3 considers the cultural dependence and inevitable scope variation of such a concept of professional responsibility, while sections 4 and 5 resolutely shift from ‘conceptual rescue’ to outlining the case for much-needed reform. Section 4 outlines the ethical implications of a vulnerability-based account of professional responsibility by calling for an ‘ethics of attention’ (in contrast to an ‘ethics of care’, which it need not entail). Putting ‘empathy in its proper place’, a professionalism driven by such an ethics of attention would be sceptical of any attempt to capture *a priori* one’s relevant professional obligations. Because these obligations can only be delineated after careful engagement with the person seeking professional services, section 5 examines the potential inherent in the courts’ articulation of a ‘duty to consult’. Deemed to be generated by the right to respect for private and family life under article 8 of the European Convention on Human Rights (ECHR), the ‘duty to consult’ established in *Tracey*^4^ was found to ‘involve a discussion, where practicable, about the patient’s wishes and feelings’^5^ (rather than the mere transfer of information). The need to expand the scope of such a duty not only beyond its current do-not-resuscitate orders confines but also beyond healthcare is but one aspect of the legal consequences that flow from a vulnerability-based redelineation of professional responsibility. It is also the most important.

In many areas of healthcare, the reform entailed by such expanded ‘duty to consult’ would not be nearly as dramatic for the practitioners as they would be for the courts, who would have to shift their focus from the nature of the professional’s expertise to the characteristics of the lay-professional relationship. In the relatively neglected domains of legal (section 5A) and educational (section 5B) malpractice, however, the consequences of such ‘duty to consult’—were it to be introduced for any lay-professional relationship characterised by the sense of self vulnerability mentioned above—would be felt at all levels. Aside from its impact on lawyers, educators, clients, pupils and their parents, the courts would, at long last, have to put an end to what Herring refers to as their ‘binary approach’^6^ to vulnerability.

## 2. Sense of Self Vulnerability: Neither Corporeal nor Epistemic

The ‘inherent’ forms of vulnerabilities that are central to the way our legal (and ethical) understanding of professional responsibility has been structured so far mostly take two forms: aside from the fact that we are fragile, embodied creatures, there is also the fact that none of us have either the time or the epistemic resources needed to competently navigate all aspects of today’s world. That means we frequently rely on expert service providers (some of whom are deemed ‘professional’). This section contrasts these two ‘inherent’ forms of vulnerability with a situational, ‘sense of self’ vulnerability which has not, so far, played as important a role as it should when it comes to delineating the obligations that stem from *professional* responsibility.

### A. Inherent Forms of Vulnerability: Epistemic and Sometimes Corporeal

The knowledge asymmetry that prompts the need for expert service providers often leaves those resorting to such experts in a position of epistemic vulnerability, which is exacerbated by the difficulty inherent in establishing an expert’s credentials. Endeavouring to climb some unknown mountain without resorting to the expertise of a mountain guide is reckless. Embarking upon a mountain expedition with a supposed mountain guide who turns out to be a fraud is perilous too. Much the same goes for repairing cars and scuba-diving. In all of those cases, our epistemic vulnerability is aggravated by the fact that what Fineman describes as the ‘universal’^7^ vulnerability we all share as ‘enmattered’ beings is also at stake.

Given the public interest in a wide range of expert services being delivered safely and reliably, a series of safeguards and obligations^8^ are typically put in place to address these ‘inherent’ forms of vulnerability (the corporeal aspect is only sometimes compounding the epistemic dimension). They govern, among other things, experts’ accreditation and the remedies available should experts’ services prove wanting. They may also impose some risk disclosure obligations (to precede a scuba-diving expedition, for instance).

As a sub-group within the wide range of expert service providers, most ‘professional services’^9^ will also entail some degree of epistemic vulnerability, as well as, in some cases, corporeal vulnerability. When considered solely through the lens of this dual vulnerability, there is no reason why the provision of ‘professional’ services should entail a different kind of responsibility from that of all expert service providers. On this line of reasoning, accreditation mechanisms combined with risk disclosure obligations may be deemed an appropriate response to the vulnerabilities at stake, whether on the side of a mountain or in an operating theatre or court antechamber. The epistemic (and, in some cases, corporeal) vulnerability that is meant to be addressed through informed consent sheets is just as significant in mountainside expeditions as it is in medical intervention contexts.

Yet most people will have the intuition that there is an important difference between the choices that a healthcare, legal or education professional is meant to empower and those at stake in a mountain expedition. Unlike the latter, both the substance and the modalities of the former will impact the extent to which a patient, client or pupil is capable of retaining a sense of ‘authorship’^10^ over the way she projects herself, both socially and through her body. The next section highlights the extent to which this context-dependent, ‘sense of self’ vulnerability silently informs intuitions that are key to our having retained a distinct concept of *professional* responsibility, which reaches well beyond healthcare, from education to many legal services.

### B. Sense of Self Vulnerability in the Provision of Professional Services: Both Situational and Relational

Of those expert services whose safe delivery is in the public interest, healthcare,^11^ some legal and financial services and education stand out because their provision is often concomitant with a type of vulnerability that is not reducible to ‘mere’ epistemic vulnerability. Nor is it equivalent to the ‘universal’, corporeal vulnerability described by Fineman. The latter is at stake in the provision of a wide range of expert services, from car mechanics to healthcare providers, via mountain guides. Yet the services provided by a mountain guide do not typically bear on the continued development of those interests and concerns that are closest to our sense of self. With educators, by contrast, the materials with which we develop a sense of self are collected and given shape. The inherent vulnerability triggered by this phase of self-development can be either moderated or intensified, depending on the educator’s professional stance,^12^ just as the vulnerability that stems from events such as prosecution, loss of employment, grave illness or divorce can. All these events have in common the fact that they can radically—sometimes overnight—transform one’s social identity,^13^ so that one is at risk of becoming ‘the person accused of rape’ or ‘the person who has cancer’. In that sense, such circumstances give rise to what Mackenzie would refer to as a ‘situational’ (rather than inherent) vulnerability:

Situational vulnerability is context specific and is caused or exacerbated by social, political, economic, or environmental factors; it may be short term, intermittent, or enduring. For example, a person who has just lost his job is situationally vulnerable. This vulnerability may be short-lived if he has educational qualifications and skills that are in demand in the marketplace.^14^

If she is to challenge the profound transformation triggered by such events (or retain a sense of authorship over the transformations inherent in the process of growing up), the person resorting to professional services will need to retain the ability to meaningfully contribute to the way she projects her sense of self. To have such a sense of self indeed requires that there be, to a minimal degree, some movement to and fro between the process of definition of our ‘self’ *from without* (natural events and human encounters) and *from within* (the way we appropriate these events and encounters). This to-and-fro movement is never easy. In the circumstances described above, a professional’s stance can have a considerable impact on how arduous it becomes. As such, the vulnerability at stake is both situational and relational: those lay-professional relationships that are concomitant with circumstances such as illness, prosecution or job dismissal can all too easily reinforce, rather than alleviate, the deep-rooted disconcertion that stems from such events.

Even when the knowledge asymmetry is made less considerable (thanks to internet resources and/or information sheets), the lay person may be left with little or no possibility to ‘input’ into those events (both as they unfold and in their aftermath). This isolation may be the result of routine adherence to formal procedures,^15^ or what Fricker describes as ‘testimonial injustice’: ‘a speaker suffers testimonial injustice just if prejudice on the hearer’s part causes him to give the speaker less credibility than he would otherwise have given’.^16^ The negative stereotypes which the ‘hearer’s prejudice’ stems from are commonly associated with certain illnesses or disabilities, but they can also be rooted in less tangible forms of social stigma. Most importantly, the current delineation of professional obligations solely by reference to ‘inherent’ forms of vulnerability—such as the disclosure obligations meant to address our epistemic vulnerability^17^—can have the insidious effect^18^ of undermining the extent to which the lay person feels she has a voice that matters within a two-way consultation. In all such cases, the lay person is precluded from meaningfully contributing to the delineation of the events or circumstances affecting her, thus making it particularly difficult to retain the minimal degree of to-and-fro movement described above.

Of course, the circumstances that prompt recourse to a professional need not be as tragic as those discussed above. Many of those circumstances will be rather mundane and unlikely to ever imperil the continued construction of one’s ‘sense of self’. In such cases,^19^ the responsibility at stake is no different from that of other expert service providers. The resulting, shifting boundaries of professional responsibility are discussed in the next section.

## 3. The Scope of Professional Responsibility: Socio-cultural Dependence

Today, there are many occupations deemed ‘professional’ which never encounter the particular vulnerability described earlier. Think of pathologists, or lawyers mostly representing corporations or organisations of various kinds, for instance. Outside of the legal and medical professions, many occupations that are categorised as professions similarly do not give rise to the very particular type of vulnerability I have highlighted: scientists, most engineers, urban planners … The list goes on, and includes all sorts of occupations whose certification procedures, codes of ethics, etc are deemed to signal their ‘professional’ status.

This section discusses the discrepancy between the scope of the professions as a sociological concept and the scope of the specific, professional responsibility as it is delineated in this article. I argue that those occupations which typically encounter the particular kind of vulnerability described in the previous section give rise to a kind of responsibility that is qualitatively different from the responsibility of expert service providers in general. Importantly, this qualitative difference does not diminish in any way the importance of one’s ‘generic’ responsibility as expert service provider: the consequences of carelessness in the provision of such expert services can be disastrous, from loss of life to long-term damage to the social fabric. What this vulnerability-based account does is highlight the particular nature of the responsibility that characterises a significant chunk of the professions, and the specific demands that stem from it.

### A. The Scope of the Professions as a Sociological Concept

In its adjectival form, the term ‘professional’ is easy enough to define as the opposite of amateurism. Things get more complicated when it comes to grasping what is entailed by the plural noun ‘the professions’. This is in part because of its loose colloquial use today to cover any occupation that has been professionalised by the introduction of a code of ethics, formal training and accreditation. Reference to these formal characteristics as a lowest common denominator defining the professions tends to go hand in hand with a functional analysis. The latter was key to the birth of the sociology of the professions as a field. One of the foundational studies by Carr-Saunders and Wilson highlighted the professions’ role in ‘preserv[ing] and pass[ing] on a tradition’ to resist the ‘crude forces which threaten steady and peaceful evolution’.^20^ This functional analysis gave rise to a dominant tradition within the sociology of the professions, which aims to capture the professions’ key, differentiating characteristics.^21^ Based on the assumption that professionalism is best understood as an inherent characteristic of particular occupations, these studies typically emphasise their formal self-regulation and training programme, a commitment to a rather vague notion of ‘public service’, the nature of their knowledge base, etc.

Today, calls to professionalise occupations such as management in a bid to ‘remedy […] the crisis of legitimacy now facing American business’^22^ typically proceed on the basis of a point-by-point comparison with other professions. In a move that illustrates the influence of the above ‘trait-based’ analysis of the professions, the authors of ‘Is Business Management a Profession?’, for instance, refer to four traditional ‘bona fide’ professions criteria: (i) a widely accepted, theoretically informed body of knowledge; (ii) certification procedures; (iii) commitment to the public good; and (iv) a code of ethics with compliance procedures. Even though management pales in comparison to the medical and legal professions in relation to all of those four criteria, the authors emphasise that calling for the professionalisation of management could bring about substantial improvements in all four respects, which would benefit society as a whole.

This reference to professionalisation as a tool for social engineering is far from new. It may be said to be concomitant with the birth of the professions as an institution. Such social engineering can take a more or less conservative tone: in contrast to Carr-Saunders and Wilson’s emphasis on the professions’ role in ‘preserv[ing] and pass[ing] on a tradition’,^23^ contemporary calls for professionalisation tend to be less conservative, but the functional approach is similar. In a bid to dispute such trait-based and functionalist accounts, Johnson^24^ successfully denounced professionalism as a means of reinforcing and controlling the power concomitant with being the exclusive provider of a particular kind of service. In a related critique, Freidson highlighted the stakes inherent in the professions’ retaining control over ‘the social and economic methods of organising the performance of their own work’.^25^ Far from necessarily effecting desirable change, calls for professionalisation—such as the management example described above—all too often end up lending a veneer of respectability while at the same time resulting in less (rather than more) accountability to the public.

Power struggles—themselves determined by a complex web of political and cultural factors—will dictate whether such calls for professionalisation succeed in justifying the growth of the sphere of the professions as a sociological concept. To determine whether or not a particular occupation (or members thereof) should be held to specific, professional responsibility standards, by contrast, one needs to consider whether it encounters the specific kind of vulnerability described in section 2. Answers to this question will vary, both geographically and across time; this is discussed in the following section.

### B. The Shifting Scope of a Vulnerability-Based Concept of Professional Responsibility

The scope of professional responsibility as it is defended here will necessarily be the object of ongoing rearticulation and contestation. As one moves away from ‘central cases’ (such as psychiatrists, CAB advisers, criminal or family lawyers and teachers) to occupations that only occasionally encounter the vulnerability described in section 2, new ‘borderline cases’ will emerge as the nature of existing and emerging professional work evolves. Think of ‘bankers’:^26^ today, fewer and fewer bankers have anything to do with private individuals, and fewer still are ever confronted with circumstances where their professional stance may either reinforce or alleviate the specific, lay vulnerability described above. Except for the latter cases, the nature of most bankers’ responsibility ends up being no different from that of corporate lawyers or expert service providers in general.

The scope of professional responsibility will also vary historically and across societies: in a less materialistic society (one that does not foster a strong connection between one’s sense of self and wealth), the responsibility of client-facing bankers and financial advisers may not be deemed any different from that of other experts. In societies where the development of one’s sense of self is widely acknowledged to be dependent on a spiritual dimension, by contrast, spiritual advisers and/or religious representatives will have the same type of responsibility as that of healthcare providers or educators.

This explicit cultural dependence stands in sharp contrast to public-good^27^ or dignity-based accounts of professional responsibility. The superior explanatory power of a vulnerability-based account is particularly evident when considering the way in which some occupations may cease to give rise to the particular, professional type of responsibility as I have defended it, while others may come to acquire it. What if medicine was so advanced that it only ever required at most one ‘day off’ in order to successfully cure or address any ailments? In such a desirable scenario, one may argue that the responsibility of healthcare providers would be no different from that of other experts, since being ill would not prompt the very specific type of vulnerability discussed above.

Concomitantly, contemporary technological advances deployed in domains not traditionally associated with the professions may be argued to have *expanded* the domain of professional responsibility. Today, it is near impossible to go about one’s life without leaking data on a daily basis, even for those remaining mostly ‘offline’.^28^ Over time, a detailed picture of who we are and what motivates us emerges,^29^ allowing our lives to be dissected to an unprecedented degree.^30^ This makes us vulnerable. This vulnerability can be exploited in a way that compromises our ability to retain the minimal sense of ‘authorship’ described earlier, just like the vulnerability of the elderly, the ill or the prosecuted can be overlooked with similar effects. Without the proposed redelineation of professional responsibility, it is difficult to explain why the vulnerabilities triggered by such technological advances can be said to transform the nature of the responsibility of those in the business of harvesting or processing our personal data. Since the services provided by the latter are concomitant with a vulnerability that is distinct from the ‘inherent’, epistemic version, their responsibility ought to entail obligations that are distinct from those of all expert service providers.^31^ While section 5 emphasises the importance of one legal obligation in particular—the duty to consult, the next section (4) delineates the role played by an ‘ethics of attention’.

## 4. Calling for an ‘Ethics of Attention’

Many will readily concede that the circumstances giving rise to the need for professional services are, in some domains at least, frequently characterised by the ‘sense of self’ vulnerability described earlier, yet question whether this should have any impact on the delineation of professional responsibility and its concomitant obligations. Before considering the case for a legal ‘duty to consult’ (section 5), this section delineates the ethical consequences of such a vulnerability-based concept of professional responsibility. It does so by calling for an ‘ethics of attention’ which focuses on the specificities of each lay-professional encounter, rather than the responsible agent’s capacities (or expertise).

An endeavour to deny any responsibility for the ‘sense of self’ vulnerability described earlier could argue that there is only so much a professional can control: yes, a professional should be held to stringent safety standards and yes, she should make sure that her client/patient/pupil is equipped with all the information relevant to the situation or decision at stake. But no, she should not be expected to engage with each of her clients/patients/pupils (including appropriate representatives) in such a way as to understand the kind of person they wish to become and the extent to which the situation at stake impacts upon those aspirations. As a professional, she cannot be expected to be in control of the psychological and social aspects that underlie the situation that triggered the need for her expertise.

From a theoretical perspective, the above line of argument is in part facilitated by the dominant conceptualisation of moral responsibility by sole reference to the responsible agent’s capacities. This model typically highlights the capacities absent in those we would deem non-responsible as grounds for responsibility: features such as control^32^ or rationality^33^ tend to dominate such accounts. On this line of reasoning, a person who does not have a minimum degree of control^34^ over the impact of her actions (or attitude) cannot be held responsible. This focus on the responsible agent’s characteristics—rather than the nature of the expectations^35^ structuring her relationships with others—has served the professions rather well. Johnson^36^ successfully denounced professionalism’s self-serving rhetoric—whereby it is the professions’ superior knowledge and selfless integrity that warrants non-interventionism in the face of professional discretion—decades ago. Yet the courts are only just starting to see past knowledge asymmetry as the lay-professional relationship’s defining characteristic; this legal aspect will be elaborated on in the next section.

For now, it should be enough to point out the extent to which the—so far dominant—conceptualisation of responsibility by reference to the responsible agent’s characteristics reflects power dynamics^37^ designed to shift attention away from the moral demands entailed by each lay-professional relationship. Given the wide scope of those occupations sociologically deemed part of the ‘professions’, the nature of these relationships will vary greatly. A subset, however, has one important characteristic in common: the circumstances that prompt such relationships go hand in hand with the specific, ‘sense of self’ vulnerability outlined earlier. From this particular vulnerability stem demands that cannot be met under the standard, knowledge-transaction model that underpins the provision of all expert services.

To be receptive to these demands is incompatible with any claim to have captured what constitutes professional good practice in advance, whether in a legal, educational or healthcare setting. Such a claim is all too likely to shield the professional from ever having to see past ‘the usual person in the usual place’^38^ and adopt what Dewey referred to as a spirit of ‘enquiry’.^39^ From a theoretical perspective, Socratic scepticism towards any claim to ‘know *a priori*’ what constitutes excellence in the domain of virtue (in professional contexts or otherwise) is at the heart of what is sometimes called the ‘anti-theory’ movement in ethics. This latter, anti-theoretical movement is united against any understanding of moral judgments that ‘can be thought of as consequences of applying abstract principles to moral problems in an almost computational way, giving a procedure for deducing the morally correct answer in any given circumstances’.^40^

Importantly for our purposes, such an anti-theoretical stance goes hand in hand with what might be called an ‘ethics of attention’.^41^ Driven by a concern with understanding ‘the other’s point of view as potentially expressive of a whole way of being’,^42^ this ‘ethics of attention’ aims to train the capacity at the same time to see details, and to grasp the overall sense, the ‘point’ in the lives of people. Putting ‘empathy in its proper place’,^43^ a professionalism driven by such an ethics of attention would emphasise ‘the detachment required to replace the personal subjectivity of a merely sympathetic emotional reaction with an objective response based on empathy, evidence and careful deliberation’.^44^

This emphasis on the responsibilities that are forged on the basis of *connections* between people points at a shared methodological lineage with the ethics of care most famously put forward by Gilligan.^45^ Despite these affinities, this ethics of attention is nevertheless usefully distinguished from an ethics of care. Why? Because an ethics of attention remains agnostic about the substantive presuppositions that typically underlie an ethics of care, whether these presuppositions are translated in terms of benevolence as a virtue or as a disposition. While an ethics of attention is compatible with the latter, substantive positions, it is also compatible with deontological, Kantian, Confucian or other stances. What matters, in a nutshell, is the relational focus, and a commitment to unpack the responsibilities that stem from the specificities of each relationship.

## 5. The Case for a Legal ‘Duty to Consult’

Another way of deflecting the demands that stem from the ‘sense of self’ vulnerability outlined earlier is to acknowledge the specific vulnerability concomitant with many lay-professional relationships while at the same time limiting its consequent scope to moral obligations only. This line of argument would emphasise the extent to which addressing the said vulnerability entails a qualitative shift in attitude (rather than ‘act obligations’) that is not easily translatable in legal terms. Any legal translation endeavour, on this line of reasoning, would prove self-defeating,^46^ for what the lay person needs is genuine, personal engagement,

and precisely not because one has a legal claim to it. Moreover, attempts to conceptualize human needs and vulnerability in the domains that support self-trust and self-esteem in terms of rights that can be individually possessed are strained beyond plausibility: it is particularly clear here that these are fundamentally relational circumstances. Knowing oneself to be the object of very personal concern or having the sense that one’s undertakings are considered worthwhile—these are not matters that one person has in independence from a relationship. They are emergent properties of relationships of a certain sort.^47^

While the above quote was not written with professional relationships in mind, it provides a useful backdrop against which to judge the desirability of any legal obligations formulated in answer to the situational vulnerability inherent in many lay-professional relationships. The latter obligations can impose a process, rather than be result-based. As with any process-based obligations, there is always a risk that these are merely paid lip service to. In the absence of concomitant ethical awareness, genuine engagement with the lay person is likely to be compromised. In many respects, the situation is similar to the legal obligations that pertain to non-discrimination obligations in employment relationships. Without some underlying ethical awareness, what these obligations can achieve is limited, yet important.

Whether it be in the domain of employment or lay-professional relationships, for these process obligations to bear fruit particular attention needs to be paid to the power imbalances underlying those relationships.^48^ In the professional domain, the complex^49^ evolution of power dynamics within education and legal practices have not received nearly as much attention as those within healthcare, even though what is at stake is ultimately no different. Education attainment, health and legal status heavily condition the extent to which we are in a position to develop a sense of authorship^50^ over the way we project ourselves, both socially and through our body. Our reliance on professional services in such circumstances is necessarily concomitant with a vulnerability that need not be actively exploited^51^ to become pathogenic.^52^ Merely ignoring such vulnerability will corroborate conventional assumptions about expected roles, in turn diminishing the extent to which the lay person is likely to seize any opportunity to engage in constructive conversation (if there ever was one).^53^

In the above context, imposing a legal obligation to abide by a process designed to facilitate a two-way—rather than one-way—conversation may be deemed a plausible—if necessarily imperfect—way of counteracting otherwise blunted reactive attitudes. In the domain of healthcare, a ‘duty to consult’ (rather than merely informing) has recently been formulated by UK courts in end-of-life care contexts. Section 5A outlines the developments that have led to such a ‘duty to consult’ within healthcare, while section 5B argues that it is time such ‘duty to consult’ be extended to all lay-professional relationships that are characterised by the sense of self vulnerability outlined earlier.

### A. The Emergence of a ‘Duty to Consult’ within UK Healthcare

The courts’ articulation of the standards by reference to which the quality of the doctor–patient relationship may lend itself to judicial oversight has long been dominated by a narrow concern to address epistemic vulnerability through information disclosure. The drawbacks of such a narrow, epistemic focus are evident in the leading case in this domain: *Montgomery v Lanarkshire.*^54^ The latter has been welcomed by many commentators as a watershed decision^55^ because it restricts the scope of the courts’ otherwise broad deference to clinical discretion: liability for failure to disclose an intervention’s risks or potential side effects is not to be assessed solely by reference to what constitutes reasonable medical practice according to peers, or the so-called Bolam Test. In other words, risk disclosure obligations are no longer deemed to fall under clinical discretion. In the *Montgomery* case, this means that the adequacy of an obstetrician’s decision not to inform her diabetic patient—Nadine Montgomery—of the risk of shoulder dystocia (and the resulting risks of hypoxic injury for the child) can be assessed by the court without having to defer to peer judgment.^56^ In reaching this decision,^57^ the Supreme Court goes against the lower courts’ taking into account the fact that Nadine Montgomery was an educated, well-advised patient whose relationship with her doctor was based on what is best described as a *conversation* about the planned delivery (and hence different from merely being at the receiving end of ‘disclosed information’). Instead, the Supreme Court notes that ‘the social and psychological realities of the relationship between patient and her doctor’ mean that ‘few patients do not feel intimidated or inhibited to some degree’.^58^

For the Supreme Court, the fact that ‘few patients do not feel intimidated’ warrants an obligation to disclose all the ‘material risks involved in any recommended treatment, and […] any reasonable alternative or variant treatments’.^59^ What counts as a ‘material risk’ hinges upon both what a ‘reasonable’ patient *and* what the ‘particular’ patient may find significant. This two-sided materiality test requires of healthcare providers convoluted mental feats. While each encounter with a patient is meant to remain unimpeded by *a priori* categorisations that would stand in the way of acknowledging each patient’s singularity, the objective, ‘reasonable patient’ constraint seemingly calls for the opposite. In this context, the risk is that defensive, blanket disclosures^60^ based on a conservative understanding of the ‘reasonable patient’ might take over and transform Montgomery’s well-intentioned intervention into a ‘pathogenic’ response to vulnerability: ‘rather than enabling a person’s autonomy, they compound this sense of powerlessness and loss of agency and render her susceptible to new or different harms’.^61^

Had the Supreme Court chosen to interpret the fact that many patients feel ‘intimidated or inhibited’ as indicating a type of vulnerability that goes beyond epistemic aspects, it may have considered a move away from the formal information disclosure approach, as the Court of Appeal did in *Tracey.*^62^ In the latter, the Court of Appeal articulates a ‘duty to consult’ as an answer to the perceived lack of openness (on the part of doctors) about the fact that a decision was being made about the appropriateness of cardiopulmonary resuscitation. The Court found that article 8 ECHR incorporated a right, on the part of a patient and her family (which was later extended to carers^63^), to be consulted and appropriately involved in end-of-life decisions.^64^

Because it shifts emphasis from mere information disclosure to an obligation to *engage* with the patient, such a duty to consult^65^ is certainly much better suited to the situational, ‘sense of self’ vulnerability outlined in section 2. Whereas information disclosure may mean that a patient is *free*^66^ to make choices in a minimally informed^67^ manner,^68^ the kind of engagement required by a duty to consult is meant to foster a very different understanding of autonomy, one that is far more concerned with the challenge inherent in preserving a sense of authorship than it is with freedom of choice. The institutional and legal challenges inherent in articulating such a duty to consult beyond cardiopulmonary resuscitation contexts are unpacked in detail elsewhere.^69^ Of particular relevance is the Mental Health Act’s ongoing review and consultation process, which may constitute a unique opportunity for such extension.^70^ For the purposes of this article, what matters is to note the complementarity of such a duty to consult with the vulnerability-based concept of professional responsibility outlined in section 2. This complementarity lends weight to the need to expand the reach of this ‘duty to consult’ not only beyond the confines of cardiopulmonary resuscitation decisions, but also beyond healthcare.

### B. A ‘Duty to Consult’ beyond Healthcare?

Discussions of the kind of communication obligations a professional has to meet in order to live up to her responsibility have been dominated by those specific to the healthcare context. Judicial intervention when it comes to such communication obligations has been limited in ambition within legal practices (trailing behind developments in healthcare) and non-existent within education. This section critically assesses the way the courts have so far delineated—or refrained from delineating—the communication obligations of those two professions as part of a wider standard of care. In both cases, the problem stems from the courts’ enduring preoccupation with the type of expertise yielded by such professionals (and the extent to which it lends itself to judicial oversight), rather than the characteristics of the lay-professional relationship. A shift of focus to the latter would make it apparent that the former is neither here nor there when it comes to delineating what should ideally be a ‘duty to consult’. Such a duty would be applicable in any circumstances where the situational vulnerability outlined in section 2 is present.

#### (i) Communication obligations within legal practice

In the legal domain, the dominant view^71^ places much emphasis on remedying the knowledge asymmetry^72^ between client and lawyer. This concern with the provision of sufficient information to enable ‘people’ to ‘make as many decisions as possible for themselves, exercising free will’,^73^ proceeds in large part from a reaction to what is perceived as some excessive, ‘traditional’ paternalism.^74^ This reaction is most explicitly echoed in Boon and Levin’s arguing that those seeking legal advice ‘should not be seen as passive recipients of advice or assistance, but as consumers’.^75^ This candid reference to those seeking legal advice as ‘consumers’ has the merit of highlighting the problematic, minimalist understanding of autonomy that informs the so-called ‘standard conception’ of the lawyer–client relationship.

In contrast, Dinerstein defends the need for ‘shared decision-making responsibility’ and ‘mutual participation’ in a ‘client-centred’^76^ model that bears striking resemblance to the ‘shared-decision making’ model within healthcare.^77^ Within legal practice, there are different ways^78^ of articulating what is entailed by such ‘shared decision-making’. While it does presuppose that lawyers ‘take into account the real-life situation of their clients, including all their needs, desires and interests’,^79^ such a concern with the particular circumstances of each client is compatible with two very different understandings of underlying professional obligations. One may, on the one hand, perpetuate the ‘free will’ archetype, and deem such attention to a client’s particular circumstances to be necessary in order to ensure that a client is genuinely in a position to ‘make decisions for herself’^80^ by transferring relevant information. Alternatively, one may seek to ascertain whether the circumstances that prompt recourse to a lawyer go hand in hand with the situational vulnerability described earlier, and consequently assess the need to go beyond the mere transfer of information (if not legal, then as an ethical obligation).

Somewhat predictably (given access to justice challenges), the pertinent ‘legal practice’ case law concerns lay-professional relationships that are *not* characterised by such situational vulnerability. The latter aspect was neither here nor there for the courts: the central question in *Barker v Baxendale-Walker*^81^ was whether conveying the relevant risks could or should be distinguished from the substantive legal advice^82^ given by a (tax) lawyer, Mr Baxendale-Walker. The latter put together some aggressive tax evasion scheme that was meant to save his client, Mr Barker, millions in capital gains tax liabilities. When the scheme failed, Mr Barker claimed that Mr Baxendale-Walker’s failure to warn him of the risk that HM Revenue & Customs might take a contrary view was negligent. What is particularly interesting (and problematic) about this case are the arguments relied on by Lady Justice Asplin to distinguish *Montgomery* (whose relevance outside the clinical context was asserted by Mr Justice Kerr in *O’Hare v Coutts*^83^).

Asplin LJ refers to *Montgomery’s* differentiating between the doctor’s ‘advisory role’ versus those aspects of medical practice that are ‘determined by medical learning or experience’.^84^ The latter include ‘considering possible investigatory or treatment options’.^85^*Montgomery* asserts that while such investigatory or therapeutic decisions remain subject to the Bolam Test, the ‘advisory role’—particularly risk disclosure—can be scrutinised directly by the court. Asplin LJ argues that the latter distinction is irrelevant within legal practice—hence the Bolam Test must continue to apply with no exception—as:

There can be no separation between the advice and any appropriate caveats as to risk. They are one and the same. The lawyer as part of the legal advice he is providing must evaluate the legal position and determine whether in all of the circumstances he should advise his client that there is a significant risk that the view he has taken about the substantive matter in question may be wrong […] It seems to me that this is a question of law and legal expertise and not a policy question.^86^

Aside from the fact that there are many in the healthcare context who would argue that the communication of healthcare risks is no less a matter of medical expertise than the communication of legal risks, the above illustrates the unfortunate consequences of Montgomery’s focus on those aspects of professional expertise that lend themselves to judicial overview versus those that do not.^87^ Not only is such attempted ‘dismemberment’ of professional expertise conceptually haphazard,^88^ it also turns the courts’ attention away from those considerations that should be shaping its delineation of communication obligations: the characteristics of the lay-professional relationship. In *Barker v Baxendale-Walker*, these characteristics did not entail any *situational*^89^ vulnerability. But what if they had?

The courts’ enduring fascination with the nature of a professional expertise, combined with what Jonathan Herring aptly describes as a ‘binary’^90^ approach to vulnerability, does not bode well for the likelihood of reforming the courts’ current, pathogenic^91^ answer to lay vulnerability. In the domain of legal practice, the only context within which the courts do impose an extra duty to ensure that a client’s decision is truly ‘hers’—above and beyond merely ‘understanding the nature and effect of the proposed transaction’^92^—are when the client’s circumstances are such that a risk of undue influence is ‘reasonably high’. Only in the latter case is there a presumption of vulnerability.^93^ Not only does the latter regime underplay the extent to which, since we are all necessarily influenced by others throughout our life,^94^ assessing whether a client’s circumstances are such that there is a high risk of *undue* influence demands the kind of engagement required by a ‘duty to consult’. This regime also underestimates the lawyer’s own position of influence, which increases dramatically in the situations of vulnerability described in section 2 (prosecution, loss of employment, divorce proceedings, etc). In this respect, the parallels with the education profession are both striking and alarming, given the courts’ particularly detached stance in the latter domain.

#### (ii) Communication obligations within the education profession

One would think that the courts’ delineation of teachers’ duty of care would be most amenable to being steered towards some kind of duty to consult, given the well-recognised need for *partnership* between a teacher and a pupil’s family. Disappointingly, the courts have so far mostly steered away from education malpractice claims (as opposed to physical injury claims). Two broad sorts of concerns are typically invoked to justify the courts’ reluctance to assess such educational malpractice claims. On the one hand, the courts have been keen to demarcate such malpractice claims as a breach of statutory duty, given the extent to which the quality of education provision is resource-dependent (and the impact that the potential for tort liability may have on such resources).^95^

The other type of consideration likely to underlie the courts’ reluctance may be framed—once again—in terms of the nature of the teaching profession’s expertise. On this front, many highlight the extent to which teacher ‘effectiveness is an elusive concept to define when we consider the complex task of teaching and the multitude of contexts in which teachers work’.^96^ Given the variable, fluid^97^ nature of teaching, David Young warns that ‘any effort to be overly prescriptive is fraught with difficulty. Regulation and regimentation in teaching need to be tempered by the subjective nature of the very act of teaching itself, lest the educational enterprise may run amok.’^98^

Those familiar with philosophical and psychological studies of the nature of medical expertise^99^ will have a sense of déjà vu. Heavily dependent on the non-cognitive grasp of a multitude of contextual features, the art of medical diagnosis may be said to be no less ‘subjective’ and patient/pupil-dependent^100^ than the art of teaching. Yet the conclusions that stem from this shared premise are starkly different. Whereas it leads to Bolam’s deference to peer professional judgment in the context of healthcare, within the education sector it leads DeMitchell and DeMitchell to boldly argue that: ‘Currently, unlike other professionals, educators are not required to perform their duties in accordance with the standard of care observed by their profession.’^101^

In the UK, the above statement is not quite correct. Unlike their American counterparts, English courts have been willing to consider educational malpractice claims when they pertain to children with special needs.^102^ Thus, in *Marr v Lambeth London Borough Council and others*,^103^ the court dismisses a negligence claim based on the failure to recognise or appropriately deal with the claimant’s special educational needs, asserting that ‘much of the claimant’s case amounted, in reality, to an action for breach of statutory duty in disguise […] Whilst many of the criticisms might have held force in a different context, they faded in an action for negligence.’

The court does recognise that ‘the existence of a statutory scheme which sought to address a particular educational need did not of itself preclude a duty of care arising’.^104^ Yet it holds that the claimant’s allegations of negligence are not made out, making explicit reference to the Bolam Test: ‘That is to say, a claimant had to show that no competent teacher, or other person who owed a duty of care to the pupil, would have acted properly in that way.’^105^

Disappointingly, at no point in its assessment of the negligence claim is the court particularly interested or concerned about the extent to which the claimant’s family was consulted with. Thus, the court notes in passing that Mr Frolish (the claimant’s teacher) had decided not to send Andrew (the claimant) to weekly remedial class for reading and writing because he judged that Andrew ‘was receiving all the support he needed [and] attendance at it would mark Andrew out as different’.^106^ There is no mention of whether or not there was any form of consultation with Andrew’s family. This lack of interest in the extent to which consultation with the pupil’s family did take place is illustrative of the wider trend highlighted in the previous section: instead of analysing the characteristics of the lay-professional relationship and the demands entailed by it, the courts’ focus is very much on peer assessment of professional judgment.

This need not be so. If one considers the two types of concerns frequently invoked to justify the courts’ reluctant stance when it comes to educational malpractice, one finds that neither type would be of particular relevance were the courts to construct a ‘duty to consult’ similar to that established by *Tracey* on the basis of article 8 ECHR.^107^ Far from necessarily entailing an unsustainable drain on stretched education budgets, a duty to consult with a pupil and her family may well prove economical in the longer term, given the established benefits of parental involvement. A ‘duty to consult’ would also provide the courts with a ‘justiciable’, formal criterion that is not affected by (and does not deny) the fluid, context-dependent nature of teaching as an art. What it would provide the courts with is a much-needed way of addressing pupils’ (and their families’) situational vulnerability. To quote Patterson: ‘the harm caused by negligence or educational malpractice may be less visible than an injured body, but many would argue that an injured, uneducated mind is far more devastating to the individual’s self-image’.^108^

## 6. Conclusion

This article starts with a bold claim: it only makes sense to keep speaking of *professional* responsibility if one is prepared to accept that there is a discrepancy between the sphere of the professions as a sociological concept and the scope of professional responsibility as an ethical and legal concept. Why? Because what calls for distinct legal and ethical obligations (compared to those that apply to all expert service providers) is a feature of the lay-professional relationship that is not necessarily present in all occupations deemed ‘professional’. That feature is the situational, ‘sense of self’ vulnerability outlined in section 2.

Distinct from the inherent, epistemic and sometimes corporeal forms of vulnerability that go hand in hand with our need to resort to expert service providers, this ‘sense of self’ vulnerability gives rise to ethical demands that cannot be met under the standard, knowledge-transaction model. That the latter has been allowed to dominate the courts’ articulation of professional responsibility standards for so long was facilitated by the general tendency to assess responsibility by reference to the responsible agent’s characteristics, rather than the nature of the expectations structuring her relationships to others. This trend has served the professions rather well. For as long as knowledge asymmetry continues to be deemed the defining characteristic of the lay-professional relationship, the courts’ delineation of obligations meant to address lay vulnerability (such as disclosure obligations) will all too frequently end up compounding the layperson’s non-epistemic, ‘sense of self’ vulnerability.

Getting the courts to shift their focus from the nature of the professional’s expertise (and the extent to which it lends itself to judicial oversight) to the characteristics of the lay-professional relationship will take time. The recent delineation of a ‘duty to consult’ in the so far narrow context of do-not-resuscitate orders nevertheless points at a particularly promising avenue for legal reform. Were it to be expanded in scope, to reach not only beyond DNACPR but also beyond healthcare, to apply to all lay-professional relationships characterised by a ‘sense of self’ vulnerability, such a duty to consult could have a transformative impact (especially in the relatively neglected domains of legal and educational malpractice).

Some might argue that the above, judicially prompted transformation cannot happen fast enough; nor do the other changes (whether they be legal, moral or institutional) called for by this article’s proposed reconceptualisation of professional responsibility. This call for reform comes at a time of upheaval in the nature of professional work. The increasingly bleak conditions within which today’s professions have to operate throw into sharp relief the widening gap between concrete work circumstances and the normative expectations that (still) structure our understanding of many lay-professional relationships. Just as the legitimacy of increasing degrees of automation is being considered within several professions, the need for new, emerging professions—such as ‘data trustees’—is also becoming increasingly clear. In this context, the need for a conceptually robust account of what, if anything, differentiates professional responsibility from that of all expert service providers has never been more urgent.

## Footnotes

1 Because of the high probability of their being in a position to alleviate or contribute to such ‘sense of self vulnerability’, psychiatrists, Citizens Advice Bureau (CAB) advisers, criminal/family lawyers and teachers could be given as examples of indisputable, ‘central’ cases of occupations always giving rise to *professional* responsibility, as narrowly defined in this article. Other occupations—such as commercial lawyers—are unlikely to give rise to such specific responsibility. This does not prevent them from being deemed members of the professions as a sociological matter of fact. This is unpacked in section 3B.

2 MacKenzie defines ‘inherent’ sources of vulnerability as ‘intrinsic to the human condition. These vulnerabilities arise from our embodiment, our inescapable human needs, and our inevitable dependence on others’: Catriona Mackenzie, ‘The Importance of Relational Autonomy and Capabilities for an Ethics of Vulnerability’ in Catriona Mackenzie, Wendy Rogers and Susan Dodds (eds), *Vulnerability: New Essays in Ethics and Feminist Philosophy* (OUP 2014) 38.

3 Martha Nussbaum, *Frontiers of Justice: Disability, Nationality, Species Membership* (Harvard UP 2006) 278.

4
*henceforth (Tracey)*

* v Cambridge UH NHSFT* [2014] EWCA Civ 822 [45]–[55].

5 Tracey [99].

6 Herring criticises law’s binary approach to vulnerability—‘either you have capacity or you don’t, if you don’t you are vulnerable, if you do you should be able to determine for yourself how you wish to live’: Jonathan Herring, *Vulnerable Adults and the Law* (OUP 2016).

7 Fineman describes this ‘universal’ vulnerability as the ‘inevitable, enduring aspect of the human condition’. It carries with it the imminent or ever-present possibility of harm, injury, and misfortune’: MA Fineman, ‘The Vulnerable Subject: Anchoring Equality in the Human Condition’ (2008) 20 Yale JL & Feminism 1.

8 Of interest is the fact that the obligations that stem from the need to address this double (epistemic and corporeal) vulnerability can be fulfilled through acts (in contrast to the attitude obligations that are required to address the ‘sense of self’ vulnerability).

9 As a matter of sociological fact, the line demarcating expert service providers from the professions will be contested: as an example, in some countries, mountain guides may well seek professional status, or be deemed professionals.

10 Mackenzie would talk of ‘autonomous agency’: C Mackenzie, ‘The Importance of Relational Autonomy and Capabilities for an Ethics of Vulnerability’ in C Mackenzie, W Rogers and S Dodds (eds), *Vulnerability: New Essays in Ethics and Feminist Philosophy* (OUP 2014).

11 Healthcare is meant to include all expert services aimed at supporting or improving our health (hence counsellors, psychologists, midwives, nurses, osteopaths, etc are all included, alongside doctors of course).

12 In that sense, the ‘inherent’ vulnerability is also relational.

13 In a related vein, Joel H Anderson and Axel Honneth, ‘Autonomy, Vulnerability, Recognition, and Justice’ in John Christman and Joel Anderson (eds), *Autonomy and the Challenges to Liberalism: New Essays* (CUP 2005) 130 refer to ‘social vulnerability’ to emphasise ‘the ways in which being able to lead one’s own life is dependent on one’s being supported by relations of recognition’. While the ‘sense of self’ vulnerability outlined here is clearly entangled with the ‘affectively laden’ attitudes they describe (in particular, self-trust, self-respect and self-esteem), it is not reducible to any of these attitudes.

14 Mackenzie (n 10).

15 These procedures can contribute to shielding the professional from the need to acknowledge the lay person, who appears merely as ‘the usual person in the usual place’: GK Chesterton, ‘The Twelve Men’ in GK Chesterton (ed), *Tremendous Trifles* (Sheed & Ward 1955).

16 Miranda Fricker, *Epistemic Injustice: Power and the Ethics of Knowing* (OUP 2007) 4.

17 These disclosure obligations typically take the form of information sheets meant to convey the relevant risks.

18 In that sense, the current delineation of professional obligations—which can end up encouraging blanket disclosure practices—may be said to be ‘pathogenic’, to borrow MacKenzie’s vocabulary: Mackenzie (n 10). See section 5A for further developments.

19 This includes, as a matter of course, all cases involving corporate clients.

20 Alexander Morris Carr-Saunders and Paul Alexander Wilson, *The Professions* (Clarendon Press 1933) 497. In a similar vein, see Durkheim’s analysis of the professions Emile Durkheim, *Professional Ethics and Civic Morals* (Routledge 1957).

21 DJ Hickson and MW Thomas, ‘Professionalization in Britain: A Preliminary Measurement’ (1969) 3 Sociology 37; Ernest Greenwood, ‘Attributes of a Profession’ (1957) Social Work 45.

22 Rakesh Khurana, Nitin Nohria and Daniel Penrice, ‘Is Business Management a Profession?’ (2005) Harvard Business School Working Knowledge.

23 Carr-Saunders and Wilson (n 20). In a similar vein, see the analysis of the professions in Durkheim (n 21).

24 Terence Johnson, ‘Imperialism and the Professions: Notes on the Development of Professional Occupations in Britain’s Colonies and the New States’ (1972) 20 The Sociological Review 281.

25 Eliot Freidson, *Professions of Medicine: A Study of the Sociology of Applied Knowledge* (Dodd, Mead & Co 1970) 185. In a similar vein, Magali Larson notably analysed the processes that led to growing professionalisation in the 19th century as a movement for the conquest of collective social status: Magali Sarfatti Larson, *The Rise of Professionalism: A Sociological Analysis*, vol 233 (University of California Press 1979).

26 Banking may be one of the professional occupations whose nature has changed most dramatically in the course of the last century, to encompass such a wide range of roles and activities that it is somewhat unhelpful to consider it under one unique umbrella.

27 ‘A manifest and demonstrable commitment to the public good is what legitimately creates the profession as a political force’: Richard Moorhead, ‘Precarious Professionalism: Some Empirical and Behavioural Perspectives on Lawyers’ (2014) 67 CLP 447, 450.

28 It is difficult to avoid leaving a data trail when engaging in ‘offline’ activities such as offline shopping (CCTV cameras) or use of public services (such as health and transport services).

29 Mireille Hildebrandt, ‘Defining Profiling: A New Type of Knowledge?’ in Mireille Hildebrandt and Serge Gutwirth (eds), *Profiling the European Citizen: Cross-Disciplinary Perspectives* (Springer 2008).

30 Hildebrandt advocates a practice of ‘agonistic machine learning’ to provide us with the ‘means to achieve effective protection against overdetermination of individuals by machine inferences’: Mireille Hildebrandt, ‘Privacy as Protection of the Incomputable Self: From Agnostic to Agonistic Machine Learning’ (2019) 20 Theoretical Inquiries in Law 83.

31 While the battery of rights and responsibilities introduced by the General Data Protection Regulation is crucial, it cannot on its own provide an adequate answer to the vulnerabilities at stake. To address the latter, I have argued elsewhere that a new profession—‘data trustees’—is needed. Acting as an intermediary between data subjects and data controllers, such data trustees would be held by a fiduciary obligation of undivided loyalty towards the data subjects (whose data rights they exercise). For more details on data trustees and the fiduciary obligations they would be held to, see Sylvie Delacroix and Neil D Lawrence, ‘Bottom-Up Data Trusts: Disturbing the ‘One Size Fits All’approach to Data Governance’ (2019) 9 IDPL 236.

32 John Martin Fischer and Mark Ravizza, *Responsibility and Control: A Theory of Moral Responsibility* (CUP 1998); John Martin Fischer, *The Metaphysics of Free Will: An Essay on Control* (Blackwell 1994); R Jay Wallace, *Responsibility and the Moral Sentiments* (Harvard UP 1994).

33 Joseph Raz, *From Normativity to Responsibility* (OUP 2011); Michael Smith, ‘Rational Capacities, or: How to Distinguish Recklessness, Weakness, and Compulsion’ in Sarah Stroud and Christine Tappolet (eds), *Weakness of Will and Practical Irrationality* (OUP 2003); Gary Watson, ‘Skepticism about Weakness of Will’ (1977) 86 The Philosophical Review 316.

34 The threshold that sets what counts as a ‘minimal degree of control’ (taking into account the inevitable luck component) is inherently an evaluative question, which has given rise to a vast literature that is outside the scope of this article.

35 Professional relationships are structured by expectations that are different in kind from what Watson describes as ‘the basic demand’: in all our interactions, we expect others to show good will and a degree of regard for us. Gary Watson, *Peter Strawson on Responsibility and Sociality* (OUP 2014) 17.

36 Johnson, ‘Imperialism and the Professions: Notes on the Development of Professional Occupations in Britain’s Colonies and the New States’.

37 In their introduction, Hutchison, MacKenzie and Oshana eloquently highlight the extent to which ‘our moral responsibility ascriptions and practices may be caught up in the dynamics of social power’: Katrina Hutchison, Catriona Mackenzie and Marina Oshana (eds), *Social Dimensions of Moral Responsibility* (OUP 2018) 19.

38 Chesterton, ‘The Twelve Men’ (n 15).

39 ‘We must be uncomfortable, troubled, pained or disturbed by the current state of affairs in order to truly inquire […] One who practises ethics gains skill and knowledge such that she has a larger arsenal at her disposal when confronting a new quandary, but the practitioner will not be “especially good” at responding to these quandaries. The more one knows, the more one is aware of how very tenuous and limited one’s knowledge is’: Kim Garchar, ‘Imperfection, Practice and Humility in Clinical Ethics’ (2012) 18 Journal of Evaluation in Clinical Practice 1051.

40 Stanley G Clarke and Evan Simpson, *Anti-theory in Ethics and Moral Conservatism* (SUNY Press 1989) 2.

41 From an intellectual pedigree perspective, such an ‘ethics of attention’ connects the classical pragmatists such as James and Dewey to figures as diverse as the late Wittgenstein, Lögstrup and Levinas.

42 Boncompagni draws a parallel between what she sees as an underdeveloped ‘ethics of attention in James’ and ‘the Wittgensteinian use of the notion of forms of life […] which helps us to focus not only on the way we live but also on the way we and others might live’: Anna Boncompagni, *Wittgenstein and Pragmatism: On Certainty in the Light of Peirce and James* (Palgrave Macmillan 2016).

43 Paul Bloom, *Against Empathy: The Case for Rational Compassion* (Random House 2017) 208 (loc). In contrast to calls to foster higher degrees of empathy among medical students, Bloom convincingly argues that an ability to demonstrate compassion while maintaining an emotional distance may be in the best interests of both doctor and patient. In the legal sphere, studies of magistrates’ and solicitors’ ‘emotional labour’ have contributed to the recent re-assessment of the role of empathy within legal practice. Sharyn Roach Anleu and Kathy Mack, ‘Magistrates’ Everyday Work and Emotional Labour’ (2005) 32 Journal of Law and Society 590; Chalen Westaby, ‘“Feeling Like a Sponge”: The Emotional Labour Produced by Solicitors in Their Interactions with Clients Seeking Asylum’ (2010) 17 International Journal of the Legal Profession 153). Westaby and Jones call for an understanding of empathy that values equally both its cognitive and emotional underpinnings: Chalen Westaby and Emma Jones, ‘Empathy: An Essential Element of Legal Practice or “Never the Twain Shall Meet”?’ (2018) 25 International Journal of the Legal Profession 107.

44 Jonathan Montgomery, ‘The Virtues and Vices of Professionalism’ in D Bhugra and A Malik (eds), *Professionalism in Mental Healthcare: Experts, Expertise and Expectations* (CUP 2011) 18.

45 Carol Gilligan, *In a Different Voice: Psychological Theory and Women’s Development* (Harvard UP 1993).

46 ‘Legal relations are a clumsy medium for securing many aspects of an individuals’ ability to develop and pursue their own conception of a worthwhile life. An adequate approach must start out from the broader range of social institutions and interpersonal contexts within which one finds the recognitional relations crucial for autonomy’: Anderson and Honneth (n 13) 139.

47 ibid.

48 In this respect, McKenna has done much to highlight the extent to which the impact of asymmetrical power relationships on our responsibility practices has been underestimated, thereby facilitating the insidious transformation of such practices: Michael McKenna, ‘Power, Social Inequities, and the Conversational Theory of Moral Responsibility’ in Hutchison, Mackenzie and Oshana (n 37).

49 The evolution of such power dynamics is complex in that the power imbalance is not always leaning the same way. In the legal domain, Whelan and Ziv have emphasised the growing influence yielded by corporate clients, who can, for instance, demand contractual terms relating to confidentiality or conflict of interests scenarios: C Whelan and N Ziv, ‘Privatizing Professionalism: Client Control of Lawyers’ Ethics’ (2012) 80 Fordham L Rev 2577. Along a similar line, Vaughan and Coe highlight the extent to which commercial clients are in a position to dictate the terms according to which a law firm is to represent them: Steven Vaughan and Claire Coe, *Independence, Representation and Risk: An Empirical Exploration of the Management of Client Relationships by Large Law Firms* (Report for the Solicitors Regulation Authority 2015).

50 Anderson’s emphasis on the need to make sure that ‘citizens have a claim to a capability set sufficient to enable them to function as equals in society’ is developed along a similar concern for autonomy as authorship: Elizabeth Anderson, ‘Justifying the Capabilities Approach to Justice’ in Harry Brighouse and Ingrid Robeyns (eds), *Measuring Justice: Primary Goods and Capabilities* (CUP 2010) 83.

51 If it is actively exploited, it becomes a form of social cruelty. On the latter and our commitment to moral equality, see Andrea Sangiovanni, *Humanity without Dignity: Moral Equality, Respect and Human Rights* (Havard UP 2017).

52 ‘Pathogenic vulnerability is a subset of situational vulnerability and functions as a way of identifying what Goodin (1985) refers to as “all those morally unacceptable vulnerabilities and dependencies which we should, but have not yet managed to, eliminate” (p. 203) … The notion of pathogenic vulnerability also helps to identify the way that some interventions designed to ameliorate inherent or situational vulnerability can have the paradoxical effect of increasing vulnerability’: Mackenzie (n 10).

53 Hutchison, Mackenzie and Oshana (n 37): ‘In both types of exchange—academia and law enforcement—those marginalized by prevailing arrangements of authority, expectations about credibility, and conventional assumptions about one’s proper “place” lack an opportunity to contribute to the interpersonal exchange.’

54
*Montgomery*

* v Lanarkshire* [2015] UKSC 11.

55 Clare Dyer, ‘Doctors Should Not Cherry Pick What Information to Give Patients, Court Rules’ (2015) 350 BMJ 1414.

56 For a more detailed, astute analysis of *Montgomery*, see J Montgomery, ‘Patient No Longer? What Next in Health Care Law?’ (2017) 70 CLP 73.

57 To support this conclusion, the Supreme Court argues that ‘the extent to which a doctor may be inclined to discuss risks with a patient is not determined by medical learning or experience’ (*Montgomery* (n 54) [84]) and therefore the ‘skill and judgment required are not of the kind with which the Bolam Test is concerned’ (ibid [85]). Disclosure decisions are highlighted as ‘attributable not to divergent schools of thought in medical science, but merely to divergent attitudes among doctors’ (Lords Kerry and Reid, ibid [84]). This attempt to tease out aspects of medical judgment that are specifically grounded in ‘medical science’ from those that are not results in a rather problematic fragmentation of expertise that is discussed in J Montgomery and E Montgomery, ‘Montgomery on Informed Consent: An Inexpert Decision?’ (2016) 42 Journal of Medical Ethics 89.

58
*Montgomery* (n 54) [58].

59 Ibid [87]. In this respect, *Montgomery* builds on Lord Bridge and Lord Templeman’s respective judgments in *Sidaway v Bethlem RHG* [1985] 1 All ER 643.

60 Along this line, Mills and Epstein note that ‘in the short run, the standard set by *Montgomery* is likely to be difficult to apply in practice. In a contractual, increasingly litigious culture, a culture wherein responsibility and blame must be privatised, doctors must lose interest in making judgments of any kind if they want to protect themselves. They will therefore be inclined to go beyond their new legal duty and disclose even the smallest risks, regardless of their relevance to the ‘reasonable patient in the patient’s position’ or to the patients themselves’. See F Mills and M Epstein, ‘Risk Disclosure after Montgomery: Where Are We Going?’ (2018) 21 Case Reports in Women’s Health 2. For a less negative take on the challenges underlying a patient-centred, clinical approach, see H Bolton, ‘Consent after Montgomery: Clinical Considerations’ in S Jha and E Ferriman (eds), *Medicolegal Issues in Obstetrics and Gynaecology* (Springer 2018) 15–18.

61 Mackenzie (n 10) 45.

62
*Tracey* (n 4) [45]–[55]. I have analysed in greater detail both the impact of and the challenges raised by *Tracey*, and the duty to consult in general, in Sylvie Delacroix, ‘At a Cross-Roads? The Courts’ Shifting Apprehension of the Vulnerability at Stake in the Lay-Healthcare Provider Relationship’ (2019) 2 Journal of Medical Law and Ethics 139.

63
*Winspear (personally and on behalf of the estate of Carl Winspear, decd) v City Hospitals Sunderland NHS Foundation Trust* [2015] EWHC 3250 (QB). Winspear for a related decision, see also *NHS Trust and others v Y (by his litigation friend, the Official Solicitor) and another* [2018] All ER (D) 167, which concerns a minimally responsive patient, after brain damage during cardiac arrest; this case discusses a duty to consult, although the emphasis is on the patient being heard.

64 Fritz and others review the problems with current do not attempt cardiopulmonary resuscitation (DNACPR) practices to outline the ‘ReSPECT’ process as an alternative. The latter is designed to ‘shift the focus from a specific decision about CPR to making personalised plans on broader emergency care and treatment’: Z Fritz, AM Slowther and GD Perkins, ‘Resuscitation Policy Should Focus on the Patient, not the Decision’ (2017) BMJ. Just in the same way as a consent process that is solely ‘information driven’ is unlikely to empower patients, a consultation which narrowly focuses on DNACPR wishes with no contextualisation within the overall goals of care has been shown to be potentially misleading and harmful: MC Beach and RS Morrison, ‘The Effect of Do-Not-Resuscitate Orders on Physician Decision-Making’ (2002) 50 Journal of the American Geriatrics Society 2057; EA Henneman and others, ‘Effect of Do-Not-Resuscitate Orders on the Nursing Care of Critically Ill Patients’ (1994) 3 American Journal of Critical Care 467.

65 Jonathan Montgomery highlights the extent to which this ‘duty to consult’ is ‘different in kind from the duties considered in Sidaway, Chester and Montgomery because it is aimed at making patients aware that decisions are being made about them and giving them the chance to take responsibility and be actively involved’: Montgomery (n 56) 102.

66 For a discussion of the interplay between liberty and autonomy arguments, and the extent to which the courts’ focus on the patient’s freedom from being left ignorant prioritises the former (liberty) over autonomy, see John Coggon and José Miola, ‘Autonomy, Liberty, and Medical Decision-Making’ (2011) 70 CLJ 523.

67 Manson and O’Neill emphasise the contrast between the ‘communicative transaction’ model of informed consent, and ‘the conduit/container model’, whose focus on ‘disclosure alone is not sufficient for […] successful informed consent transactions. By emphasising content while neglecting agency, the conduit/container model downplays, even hides, some of the distinctive norms that must be met by effective communicative transactions’: Onora O’ Neill and Neil C Manson, *Rethinking Informed Consent in Bioethics* (CUP 2007) 88.

68 C Foster, *Choosing Life, Choosing Death: The Tyranny of Autonomy in Medical Ethics and Law* (Hart Publishing 2009).

69 Delacroix (n 62).

70 The open consultation on ‘Reforming the Mental Health Act’ closes in April 2021. It is based on the Independent Review of the Mental Health Act 1983, *Modernising the Mental Health Act: Increasing Choice, Reducing Compulsion* (2018), which has attracted criticism in part because of its narrow construal of vulnerability.

71 Herring calls it the ‘standard view’: Jonathan Herring, *Legal Ethics* (OUP 2017) 127.

72 Along this line, Pepper argues that the ‘most common function of lawyers (across specialisations and areas of practice) is to provide that knowledge [which allows clients to evaluate options and makes decisions about their lives]’: Stephen Pepper, ‘Integrating Morality and Law in Legal Practice: A Reply to Professor Simon’ (2010) 23 Geo J Legal Ethics 1011, 1013.

73 A Boon and J Levin, *The Ethics and Conduct of Lawyers in England and Wales* (Hart Publishing 2008) 184.

74 As well as the power that comes with such paternalism: T Dare, ‘Mere Zeal, Hyper-zeal and the Ethical Obligations of Lawyers’ (2004) 7 Legal Ethics 24.

75 Boon and Levin (n 73).

76 Dinerstein, 541.

77 The latter explicitly informs the NICE guidance and quality standards governing medical practice in the UK: ‘Shared decision-making is when health professionals and patients work together. This puts people at the centre of decisions about their own treatment and care’: www.nice.org.uk/about/what-we-do/our-programmes/nice-guidance/nice-guidelines/shared-decision-making.

78 Some delineate the obligations entailed in such ‘shared decision-making’ within an ‘ethics of care’ framework: see notably Theresa Glennon, ‘Lawyers and Caring: Building an Ethic of Care into Professional Responsibility’ (1991) 43 Hastings LJ 1175; R Mortensen, ‘The Lawyer as Parent: Sympathy, Care and Character in Lawyers’ Ethics’ (2009) 12 Legal Ethics 14.

79 Donald Nicolson and Julian Webb, *Professional Legal Ethics: Critical Interrogations* (OUP 2000) 197.

80 Along this line, Jackson LJ asserts in *Minkin v Landsberh (Practising as Barnet Family Law)* [2015] EWCA Civ 1152, [2016] 1 WLR 1489, 38 that it is necessary to have regard to the character and experience of a client when executing the retainer. See p 30 for situations where a recipient of legal advice is deemed—or should be presumed—‘vulnerable’ (and fn 93).

81 henceforth Barker* v Baxendale Walker Solicitors and Paul Baxendale-Walker* [2018] 1 WLR 1905.

82 See also *Credit Lyonnais SA v Russell Jones & Walker (a firm)* [2002] EWHC 1310 (Ch), [2002] All ER (D) 19, 28 (Laddie LJ), asserting that the disclosure of any discovered risks arising from the execution of the retainer is not considered ‘extra-work’: a lawyer is simply deemed to be reporting back to the client on issues of concern which he/she learns of as a result of, and in the course of, carrying out express instructions.

83 Mr Justice Kerr held that ‘The reasoning in Montgomery is not, in my judgment, irrelevant outside the medical context … As in the medical context, the extent of required communication with the client should not depend on the attitude of the individual adviser’: *O’Hare and another v Coutts & Co* [2016] EWHC 2224 (QB) [206].

84
*Montgomery* (n 54) [84].

85 ibid [82].

86
*Barker* (n 81) [64].

87 For a critique of this attempted dismemberment of professional expertise, see Jonathan Montgomery, ‘Medicine, Accountability and Professionalism’ (1989) 16 Journal of Law and Society 319.

88 Jonathan Montgomery, ‘Law and the Demoralisation of Medicine’ (2006) 26 LS 185; Charles Foster and José Miola, ‘Who’s in Charge? The Relationship between Medical Law, Medical Ethics, and Medical Morality’ (2015) 23 Med L Rev 505; Zoë Fritz and Richard Holton, ‘Too Much Medicine: Not Enough Trust?’ (2019) 45 Journal of Medical Ethics 31; Dyer (n 56).

89 Because of his unfamiliarity with the intricacies of tax law, Mr Barker was inherently vulnerable. That sort of vulnerability can be adequately addressed through risk disclosure.

90 Herring, *Vulnerable Adults* (n 6) fn 3.

91 See n 44.

92
*Royal Bank of Scotland v Etridge* [2001] 4 All ER 449 (HL).

93 To add to this gloomy picture, the Law Society of England and Wales’ recently revised guidance on ‘vulnerable’ clients makes no mention of lay-professional, relational forms of vulnerability, such as the ‘sense of self vulnerability’ described in section 2: Law Society of England and Wales, *Meeting the Needs of Vulnerable Clients* (2020).

94 Chen-Wishart criticises the ‘simplistic and inaccurate paradigm of the person whose exercise of transactional freedom is being assessed’ when appraising the risk of ‘undue influence’: Mindy Chen-Wishart, ‘Undue Influence: Vindicating Relationships of Influence’’ (2012) 59 CLP 231.

95 In *X and others (minors) v Bedfordshire County Council* [1995] 3 All ER 353, the House of Lords held that it would not be reasonable (given the disruption and likely resources drain) to ‘superimpose a common law duty of care’ on education authorities considering whether special education provision should be made for children. The court reasoned that other remedies were available through specific complaints procedures.

96 James H Stronge, Thomas J Ward and Leslie W Grant, ‘What Makes Good Teachers Good? A Cross-Case Analysis of the Connection between Teacher Effectiveness and Student Achievement’ (2011) 62 Journal of Teacher Education 339, 339.

97 ‘Teaching is a complex, fluid activity [that] makes achieving any kind of consistency a challenge’: Derek Truscott and Kenneth Crook, *Ethics and Law for Teachers* (2nd edn, Nelson Education 2016) 16.

98 David C Young, ‘Educator Malpractice: A Tort Whose Time Has Come?’ (2020) 28 Education & Law Journal 297, 300.

99 Coggon and Miola (n 67); A Fiester, ‘The “Difficult” Patient Reconceived: An Expanded Moral Mandate for Clinical Ethics’ (2012) 12 American Journal of Bioethics 2; Harry Collins and Robert Evans, *Rethinking Expertise* (University of Chicago Press 2007); SG Pauker and JB Wong, ‘How (Should) Physicians Think?’ (2010) 304 JAMA 1233; Itiel Dror, ‘A Novel Approach to Minimize Error in the Medical Domain: Cognitive Neuroscientific Insights into Training’ (2011) 33 Medical Teacher 34; Itiel E Dror, ‘The Paradox of Human Expertise: Why Experts Get It Wrong’ in N Kapur (ed), *The Paradoxical Brain* (CUP 2011).

100 Young (n 98) emphasises that ‘the methods employed by a teacher are a product of personal philosophy, background, training, and in large measure, a reflection of the discipline, academic ability of the students, and the nature of the students themselves’.

101 Todd A DeMitchell and Terri A DeMitchell, ‘Statutes and Standards: Has the Door to Educational Malpractice Been Opened’ (2003) 2003 Brigham Young University Education and Law Journal 485, 485.

102
*X v Bedfordshire* (n 95) concerned a series of five linked appeals which reached the House of Lords in 1995, all putting forward claims for personal injury arising out of a statutory duty and negligence.

103
*Marr v Lambeth London Borough Council and others* [2006] EWHC 1175 (QB).

104 ibid 34. More recently, *Robinson v Chief Constable West Yorkshire Police* [2018] UKSC 4 [35] asserts that ‘there are certain circumstances in which public authorities, like private individuals and bodies, can come under a duty of care to prevent the occurrence of harm: see, for example, *Barrett v Enfield London Borough Council and Phelps v Hillingdon London Borough Council* [2001] 2 AC 619, as explained in Gorringe at paras 39–40. In the absence of such circumstances, however, public authorities generally owe no duty of care towards individuals to confer a benefit upon them by protecting them from harm, any more than would a private individual or body.’

105
*Marr* (n 103) 33.

106 ibid 69.

107 Fiduciary duties come to mind as an alternative route to the development of a wider duty to consult. In the UK, the comparatively low level of doctrinal interest for fiduciary duties and their potential as an independent ground for action in professional malpractice claims can be traced back to Lord Scarman’s dictum in *Sidaway* (n 59). This dictum unequivocally dismissed the possibility of obtaining equitable relief on the basis of a breach of fiduciary duty by a doctor. Could it be time—thanks to *Montgomery*’s calling into question the long-established *Sidaway* doctrine—to reconsider the extent to which fiduciary duties could play a role that is better suited to the kind of vulnerability delineated in this article? For an argument along this line in the context of education, see Brett G Scharffs and John W Welch, ‘An Analytic Framework for Understanding and Evaluating the Fiduciary Duties of Educators’ (2005) Brigham Young University Education and Law Journal 159; Robert P Schuwerk, ‘The Law Professor as Fiduciary: What Duties Do We Owe to Our Students’ (2003) 45 S Tex L Rev 753. In the (Canadian) legal context, see Alice Woolley, ‘The Lawyer as Fiduciary: Definining Private Law Duties in Public Law Relations’ (2015) UTLJ 285. As far as medical practice is concerned, Canada—unlike the UK—does characterise the patient–doctor relationship as fiduciary. In the United States, fiduciary duties are recognised as an independent ground for action in relation to particular obligations (such as confidentiality) in some states.

108 Arlene H Patterson, ‘Professional Malpractice: Small Cloud, but Growing Bigger’ (1980) 62 The Phi Delta Kappan 193, 193. See also ‘courts have allowed recovery for broken bones, “but not for broken minds, psyches and expectations’”: HN Janisch, ‘Education Malpractice: Legal Liability for Failure to Educate’ (1980) 38 Advocate 491, quoted by Young (n 98).

